# Myocardial CT perfusion imaging for the detection of obstructive coronary artery disease: multisegment reconstruction does not improve diagnostic performance

**DOI:** 10.1186/s41747-021-00256-8

**Published:** 2022-01-31

**Authors:** Daniel Preuß, Gonzalo Garcia, Michael Laule, Marc Dewey, Matthias Rief

**Affiliations:** 1 Department of Radiology, Charité—Universitätsmedizin Berlin, corporate member of Freie Universität Berlin and Humboldt-Universität zu Berlin, Campus Mitte, Charitéplatz 1, 10117 Berlin, Germany; 2 Department of Cardiology, Charité—Universitätsmedizin Berlin, corporate member of Freie Universität Berlin and Humboldt-Universität zu Berlin, Charitéplatz 1, 10117 Berlin, Germany

**Keywords:** Coronary artery disease, Coronary angiography, Multidetector computed tomography, Myocardial perfusion imaging, Sensitivity and specificity

## Abstract

**Background:**

Multisegment reconstruction (MSR) was introduced to shorten the temporal reconstruction window of computed tomography (CT) and thereby reduce motion artefacts. We investigated whether MSR of myocardial CT perfusion (CTP) can improve diagnostic performance in detecting obstructive coronary artery disease (CAD) compared with halfscan reconstruction (HSR).

**Methods:**

A total of 134 patients (median age 65.7 years) with clinical indication for invasive coronary angiography and without cardiac surgery prospectively underwent static CTP. In 93 patients with multisegment acquisition, we retrospectively performed both MSR and HSR and searched both reconstructions for perfusion defects. Subgroups with known (*n* = 68) or suspected CAD (*n* = 25) and high heart rate (*n* = 30) were analysed. The area under the curve (AUC) was compared applying DeLong approach using ≥ 50% stenosis on invasive coronary angiography as reference standard.

**Results:**

Per-patient analysis revealed the overall AUC of MSR (0.65 [95% confidence interval 0.53, 0.78]) to be inferior to that of HSR (0.79 [0.69, 0.88]; *p* = 0.011). AUCs of MSR and HSR were similar in all subgroups analysed (known CAD 0.62 [0.45, 0.79] *versus* 0.72 [0.57, 0.86]; *p* = 0.157; suspected CAD 0.80 [0.63, 0.97] *versus* 0.89 [0.77, 1.00]; *p* = 0.243; high heart rate 0.46 [0.19, 0.73] *versus* 0.55 [0.33, 0.77]; *p* = 0.389). Median stress radiation dose was higher for MSR than for HSR (6.67 mSv *versus* 3.64 mSv, *p* < 0.001).

**Conclusions:**

MSR did not improve diagnostic performance of myocardial CTP imaging while increasing radiation dose compared with HSR.

**Trial registration:**

CORE320: clinicaltrials.gov NCT00934037, CARS-320: NCT00967876.

**Supplementary Information:**

The online version contains supplementary material available at 10.1186/s41747-021-00256-8.

## Key points

- Overall per-patient diagnostic performance of multisegment reconstruction (MSR) of myocardial computed tomography perfusion was inferior to halfscan reconstruction (HSR) for detecting obstructive coronary artery disease (CAD).
- MSR also did not improve the diagnostic performance in terms of area under the curve in any patient subgroup analysed (CAD status, high heart rate).
- The radiation dose of MSR was higher than that of HSR.

## Background

The latest American and European guidelines [1, 2] still do not include myocardial computed tomography perfusion (CTP) for noninvasive imaging of patients with suspected or known coronary artery disease (CAD) before invasive coronary angiography (ICA). However, recent studies suggest that CTP has higher diagnostic performance than single-photon emission tomography [3] and the same as magnetic resonance imaging [4] for detecting obstructive CAD. Evidence from meta-analyses shows an added benefit of static and dynamic CTP when combined with coronary computed tomography angiography (CTA), allowing accurate anatomical and functional assessment of the coronary arteries [5, 6]. Stress imaging is generally part of CTP acquisition protocols and is performed to induce hyperaemia and thus demarcate relative myocardial perfusion defects indicative of obstructive CAD [5]. However, an undesired effect is an increase in heart rate, and CTA research suggests that the temporal reconstruction window of halfscan reconstruction (HSR) may not be short enough to accurately detect stenosis and avoid motion artefacts of the coronary arteries [7, 8] for heart rates > 65 beats per minute (bpm).

Technically, HSR uses partial scan raw data of approximately half a gantry rotation, generating a temporal reconstruction window that corresponds to that partial gantry rotation time [7–11]. A shorter temporal reconstruction window can also be generated by acquiring several heart beats (segments) and using the partial scan raw data of all segments in multisegment reconstruction (MSR). This improves the per-segment temporal reconstruction window of MSR compared with HSR by up to the same factor as the number of segments acquired [7–11] at the cost of a higher radiation dose.

A few prospective studies show that MSR improves CTA image quality of coronary arteries compared to HSR in patients with high heart rates [7, 8, 12]. In terms of diagnostic performance of CTA in the diagnostic evaluation of CAD [7, 8, 12] or myocardial function [13], available studies revealed that MSR had higher [7, 12] or the same [8, 13] diagnostic performance compared to HSR. Although, with the much faster gantry rotation speed available today, the temporal reconstruction window of HSR may be short enough to avoid motion artefacts and accurately detect perfusion defects in CTP, recent studies [14–16] still used MSR assuming superior diagnostic performance for this method in myocardial CTP as demonstrated in CTA and myocardial function. On the other hand, preferring HSR over MSR [17–19] is also not evidence-based as the diagnostic performance of MSR and HSR in myocardial CTP has not yet been compared before.

Thus, the primary objective of the present analysis was to investigate whether MSR of myocardial CTP imaging can improve diagnostic performance in the detection of obstructive CAD compared with HSR.

## Methods

### Study design and population

**Fig. 1:**
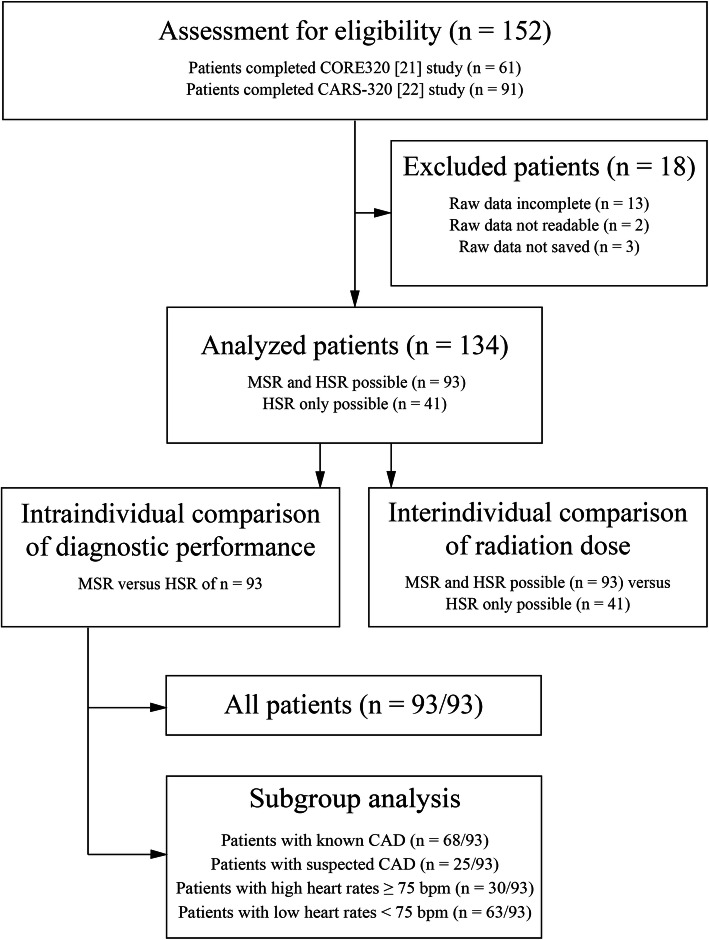
Flow chart showing the patient selection strategy for comparison of diagnostic performance and radiation dose of MSR and HSR of myocardial CTP imaging datasets in patients with suspected or known CAD. The study population for intraindividual comparison of diagnostic performance consisted of 93 of 134 patients with multisegment acquisition of stress CTP allowing both HSR and MSR. The remaining 41 of 134 patients had predefined single-segment acquisition due to heart rates < 65 bpm, and only HSR was possible. These patients served as a control group for interindividual radiation dose comparison. *bpm* Beats per minute, *CAD* Coronary artery disease, *CTP* Computed tomography perfusion, *HSR* Halfscan reconstruction, *MSR* Multisegment reconstruction

The current study is a retrospective single-centre substudy of patients prospectively enrolled in two studies: multicentre CORE320 ([20, 21], www.clinicaltrials.gov: NCT00934037) and single-centre CARS-320 ([22], NCT00967876). Both primary studies and this substudy were approved by the institutional ethics committee, and patients gave written informed consent for enrolment in the primary study and use of their data for secondary analysis. The study designs of the primary studies have been reported in detail before [20, 22] and their primary objectives along with detailed inclusion and exclusion criteria are given in Supplementary Table S1. Patient enrolment was conducted consecutively at Charité University Hospital Berlin between April 2009 and November 2011 (no randomisation or double inclusion). The inclusion criterion for the current substudy was completion of either the CORE320 or the CARS-320 study. The exclusion criterion was no availability of the full myocardial CTP raw dataset (Fig. 1). All patients underwent coronary CTA (not considered in the current analysis) and myocardial CTP followed by quantitative ICA (no randomisation). In the primary studies, a heart rate of ≥ 65 bpm was the cut-off for multisegment acquisition (vendor preset), and patients were analysed using MSR only. In the current substudy, we performed both MSR and HSR of myocardial CTP in patients with multisegment acquisition and compared the diagnostic performance of the two reconstruction techniques. Patients with single-segment acquisition were included to compile the control group for interindividual comparison of radiation dose with that of the multisegment acquisition group, as estimating radiation dose of HSR from the multisegment acquisition raw data may be inaccurate (Fig. 1). As predictive values depend on disease prevalence, and patients with known CAD of the present analysis represent a high-risk group for having obstructive CAD, we analysed the diagnostic performance of MSR and HSR separately for the two subgroups, *i.e.*, patients with known or suspected CAD. Furthermore, we analysed subgroups of patients with high heart rate (≥ 75 bpm) and low heart rate (< 75 bpm) as previous coronary CTA research indicates that the image quality of HSR decreases with heart rates ≥ 75 bpm (Fig. 1) [7, 23].

### CT acquisition, reconstruction, and reading

Details of the CTP acquisition protocols are provided in Table 1 and have been reported before [22, 24]. In brief, contrast-enhanced static CTP imaging was performed on a 320-row CT scanner (Aquilion ONE, Canon Medical Systems; former Toshiba Medical Systems) with a 350-ms gantry rotation time and a detector width of 0.5 mm. As the primary studies were planned independently from each other, and the maximum cumulative radiation dose of all CT examinations in the CORE320 study was capped to 25.5 mSv [20], the acquisition protocols of the primary studies slightly differ from each other (Table 1). In patients with heart rates < 65 bpm, a single segment was scanned (vendor preset). Patients with heart rates ≥ 65 bpm underwent a capped two-segment acquisition when they were included in the CORE320 study [24] and adaptive multisegment acquisition of up to four segments in the CARS-320 study (vendor preset) [22].

**Table 1:** Myocardial computed tomography perfusion raw data acquisition protocol

	Primary study	
Parameter	*CORE320* [24]	*CARS-320* [22]
Tube voltage (kV)	120	120
Tube current (mA)		
Body mass index^a^		
Women		
*≤ 20*	270	200
*20 ≤ 25*	300	250
*25 ≤ 30*	300	300
*30 ≤ 33*	300	350
*33 ≤ 36*	300	400
*36 ≤ 40*	300	450
Men		
*≤ 20*	300	250
*20 ≤ 25*	350	300
*25 ≤ 30*	350	350
*30 ≤ 33*	350	400
*33 ≤ 36*	350	450
*36 ≤ 40*	350	500
ECG-gated tube current modulation	off	off
Target of acquisition window (%)	85	85
Acquisition window (%)	20	20
Contrast agent		
Volume (mL), (flow [mL/s])		
Weight		
*< 60 kg*	50 (4.0)	50 (4.0)
*60 ≤ 70 kg*	60 (4.5)	60 (5.0)
*70 ≤ 80 kg*	60 (5.0)	60 (5.0)
*80 ≤ 100 kg*	60 (5.0)	70 (5.0)
*> 100 kg*	70 (5.0)	70 (5.0)
Acquisition kick-off (bolus tracking)	300 HU in the descending aorta	200 HU in the descending aorta
Number of scanned segments		
Heart rate (beats per minute)		
*< 65*	1	1
*65 ≤ 80*	2	2
*80 ≤ 118*	2	3
*118 ≤ 155*	2	4
Temporal order of CTP imaging	Stress imaging after rest imaging	Stress imaging after rest imaging
Time interval between rest and stress imaging (min)	20	20
Vasodilator in stress imaging (flow [μg/kg/min])	Adenosine (140)	Adenosine (140)

*ECG* Electrocardiogram, *CTP* Computed tomography perfusion, *HU* Hounsfield units. ^a^Calculated as $\frac{\mathrm{weight}\ \mathrm{in}\ \mathrm{kg}}{{\left(\mathrm{height}\ \mathrm{in}\ \mathrm{m}\right)}^2}$

**Fig. 2:**
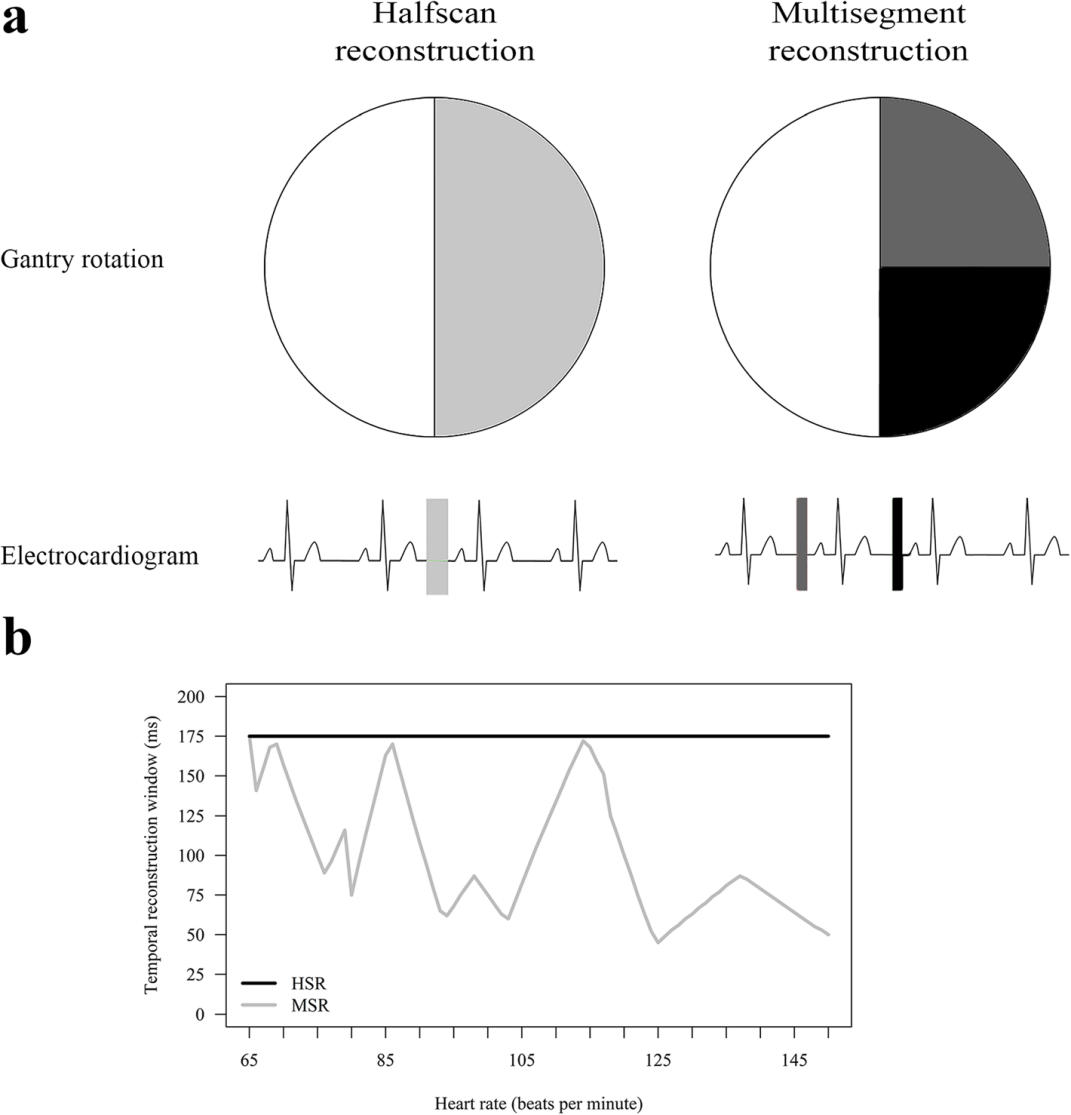
Technical background for generating temporal reconstruction windows of MSR and HSR (**a**) and their dependency on patient’s heart rate (**b**). **a** The minimum partial scan raw data needed to reconstruct a volume require half a gantry rotation (+ fan angle). HSR uses partial scan raw data acquired within one heart beat (illustrated on the left in light grey) and consequently generates a per-segment temporal reconstruction window of half a gantry rotation. MSR uses partial scan raw data of approximately half a gantry rotation acquired in at least two successive heart beats, illustrated on the right for two segments in dark grey (segment one) and black (successive segment two). The resulting minimum per-segment temporal reconstruction window of MSR can be calculated as follows: Temporal reconstruction window = gantry rotation time / (2 × number of acquired segments). Thereby, acquiring more segments improves the per (heart)-segment temporal reconstruction window of MSR compared with HSR by up to the same factor as the number of segments acquired [see references [7–11]] at the cost of a higher radiation dose [see references [8, 12]]. **b** Line graphs showing the per-segment temporal reconstruction window of MSR and HSR depending on individual patient’s heart rate for a 350-ms gantry rotation time and acquisition of up to four segments. The temporal reconstruction window of HSR is always 175 ms, whereas the temporal reconstruction window of MSR ranges from 44 to 175 ms depending on heart rate. Data by courtesy of the equipment vendor. *HSR* Halfscan reconstruction, *MSR* Multisegment reconstruction

**Fig. 3:**
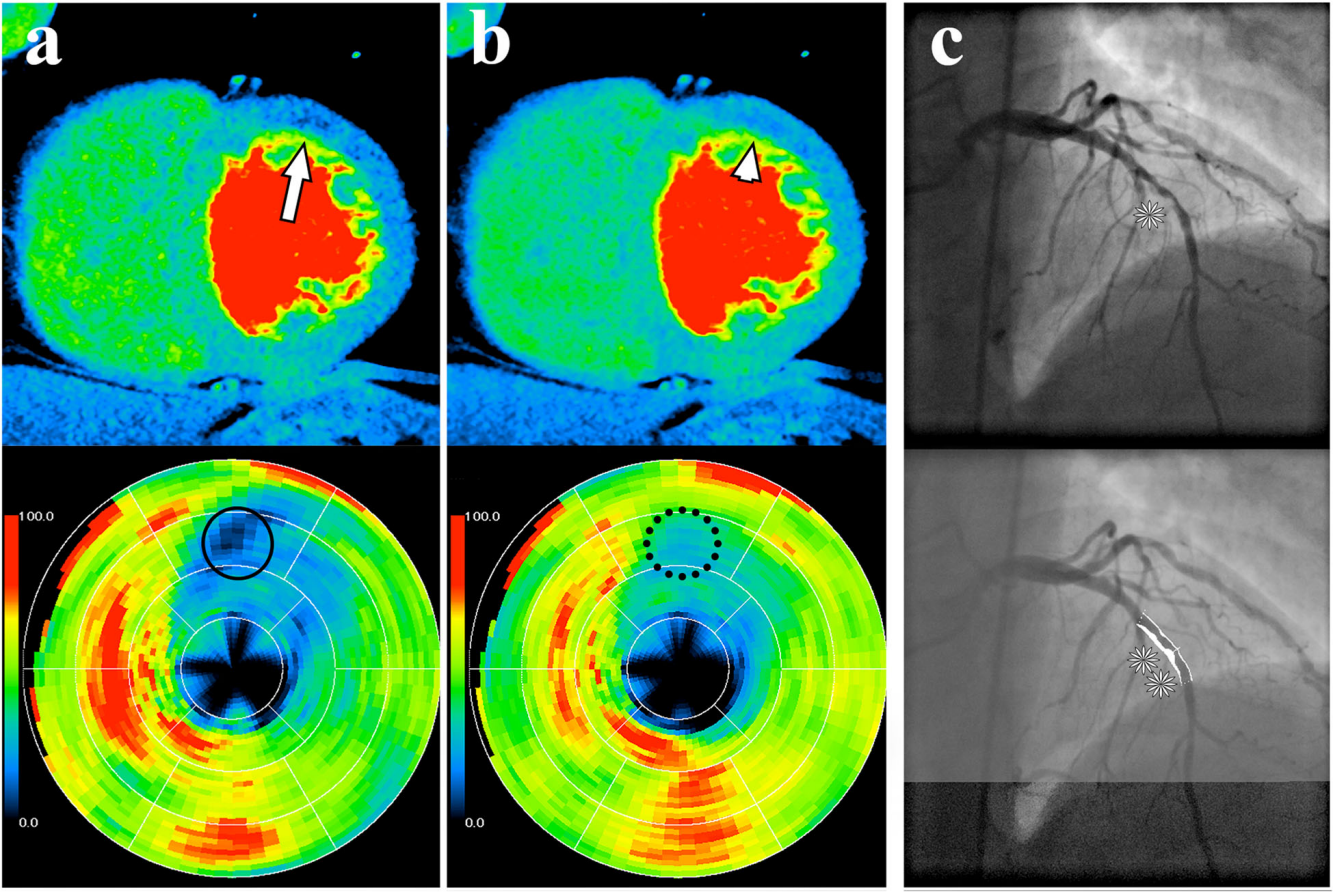
HSR and two-segment MSR of stress myocardial CTP in a 55-year-old man with suspected coronary artery disease and typical angina pectoris in comparison with invasive coronary angiography. **a** HSR shows a moderate perfusion defect in the left anterior descending artery territory (arrow), which is also suggested by moderate hypoattenuation in the corresponding area (circle) of the polar myocardial attenuation map; CTP with HSR was considered positive. **b** MSR shows only very slightly reduced perfusion (arrowhead) and only weak hypoattenuation in the corresponding area (dotted circle) of the polar myocardial attenuation map; CTP with MSR was considered negative. **c** Invasive coronary angiography reveals visually high-grade diameter stenosis (*) of the left anterior descending artery with 61% stenosis in quantitative invasive coronary angiography (**), corresponding to a true-positive CTP with HSR (**a**) and a false-negative CTP with MSR (**b**). Contrast-enhanced CTP in mid-heart short-axis view with 8-mm slice thickness and rainbow-red colour preset using a predefined window level/window width of 200/400. *CTP* Computed tomography perfusion, *HSR* Halfscan reconstruction, *MSR* Multisegment reconstruction

If multiple segments were acquired for stress CTP imaging, we performed both MSR of all available consecutive segments and HSR of the first segment, resulting in a temporal reconstruction window of 175 ms for HSR and of down to 44 ms (median 87 ms, interquartile range 69–128) for MSR (Fig. 2) when up to four segments were acquired [8–11]. HSR was consistently used for rest CTP. We applied a myocardial CTP kernel (FC03) and an iterative reconstruction algorithm (AIDR3D-standard). Further reconstruction parameters were previously described [22]. Reconstructed volumes were read on a dedicated research workstation with software version 4.71GR002, Canon Medical Systems (Otawara, Japan), using 3-mm intervals of 8-mm-thick cardiac short-axis views [22] in rainbow red colour (assigning CT attenuations to colours ranging from low attenuation (black), to intermediate (green), to high attenuation (red)) (Fig. 3). Two readers (M.R., radiologist, 10 years of experience in cardiovascular imaging, and D.P., physician, 2 years of experience in cardiovascular imaging) blinded to clinical information, coronary anatomy, and results of the CTA and reference test independently assessed images in random order. Each myocardial segment was visually judged (qualitative analysis [24]) in an intent-to-diagnose approach [25] to detect stress-induced, fixed, or partially reversible perfusion defects with additional use of the myocardial attenuation map and the transmural perfusion ratio (< 0.99) as semiquantitative parameters [22, 24]. Subsequently, each segment was classified according to readers’ rating confidence: definitely no perfusion defect, most likely no perfusion defect, possibly no perfusion defect, probably no perfusion defect, non-diagnostic, probably a perfusion defect, possibly a perfusion defect, most likely a perfusion defect, and definitely a perfusion defect. Differences between the two readers were solved in a consensus session. The American Heart Association’s 17-segment myocardial model was used [26]. Thereafter, readers were unblinded only to the patient’s individual coronary CT anatomy: perfusion defects were manually assigned to their culprit supplying arteries (right anterior descending, left anterior descending, left circumflex artery, and, if present, ramus intermedius) using the thin-sliced rest CTP images (which also served as coronary CTA dataset in the primary studies). A perfusion defect possibly caused by left main artery stenosis (50%) was assigned to one of the anatomic downstream coronary arteries. As scan timing is crucial for differentiating ischemic from normal myocardium in static myocardial perfusion during arterial contrast medium first pass [27–29], we compared CT attenuation in the left ventricle, ascending aorta, and proximal or distal descending aorta to estimate the contrast bolus phase in which the images were acquired.

### Reference: quantitative invasive coronary angiography

A diameter stenosis of ≥ 50% detected on quantitative ICA served as the common predefined reference standard [20, 22]. To consider functional relevance [16], we additionally used a ≥ 70% diameter stenosis detected on ICA as secondary reference standard. ICA was conducted in clinical standard technique [20, 22]. The degree of coronary artery stenosis was assessed by the principal investigators of these studies in an intent-to-diagnose approach. They were cardiologists with at least 5 years of experience in performing ICA and were blinded to the results of CTA and CTP. However, clinical information was available to them in a clinical setting*.*

### Statistical analysis

In contrast to the primary studies [20, 22], an additional power analysis was not conducted for this retrospective substudy. The AUC represents the readers’ confidence in the identification of perfusion defects caused by stenosis in the supplying artery. AUCs were calculated and compared using DeLong et al.’s approach [30]. To avoid overestimation of diagnostic performance, an analysis should include ‘non-diagnostic’ segments as well [25], and we think they should not be considered negative but positive (intent-to-diagnose approach). Consequently, a diagnostic confidence rating of non-diagnostic or higher for the presence of a perfusion defect was used to estimate sensitivity, specificity, and predictive values. Thereby, the threshold for a positive myocardial segment in CTP was clinically set and was not derived from AUC. The proportions were calculated with 95% confidence intervals for unclustered data [31] and compared using McNemar’s and Leisenring et al.’s methods [32]. We performed Cohen-*κ* statistics and additionally calculated raw agreement to assess inter-reader correlation. An unweighted *κ* > 0.90 was interpreted to indicate almost perfect agreement [33]. Patient characteristics, bolus timing data, and radiation doses were compared with Student’s paired or unpaired *t*-, Mann-Whitney *U*, Wilcoxon’s signed-rank, *χ*^2^, or Fisher’s exact test, as appropriate. Values of *p* < 0.050 were regarded as statistically significant. Statistical analysis was conducted by G.G. using the open-source software ‘R’, version 3.4.1 [34] with the ‘pROC_1.10.0’ [35], ‘DTComPair_1.0.3’ [36], and ‘epiR_0.9-91’ [37] packages.

## Results

### Patient characteristics

A total of 134 patients were included in this study (Fig. 1). The population for comparing diagnostic performance of MSR and HSR of myocardial CTP consisted of 93 of 134 patients with multisegment acquisition of stress CTP, allowing application of both reconstruction methods. The remaining 41 of 134 patients had single-segment acquisition and only HSR was possible. These patients served as a control group for interindividual radiation dose comparison. Detailed patient characteristics are given in Table 2. Median age of all 134 patients was 65.7 years (interquartile range 55.9–70.2); 73% were men (98 of 134) and 66% of the patients (89 of 134) presented with a previous percutaneous coronary intervention. Among all analysed patients, 60% (80 of 134) and 27% (36 of 134) were diagnosed with at least one ≥ 50% and ≥ 70% coronary vessel stenosis in the study ICA, respectively. One patient had obstructive CAD in the left main artery and corresponding perfusion defects in both the left anterior descending and the left circumflex artery. Serious adverse events occurred in two patients (one coronary dissection during ICA and one intracerebral bleeding after ICA). The median time interval between CTP and ICA was 24 h 02 min (range 1 h 35 min to 28 days 4 h 38 min).

**Table 2:** Characteristics of the 134 patients analysed in this study

Characteristic	All patients (*n* = 134)	Diagnostic performance comparison and radiation dose index group (*n* = 93)^c^	Radiation dose control group (*n* = 41)^c^	*p*-value
Age (years)^a^	65.7 (55.9–70.2)	62.9 (54.5–70.0)	67.1 (64.4–70.1)	0.013
Men	73 (98)	69 (64)	83 (34)	0.090
Body mass index^a, b^	26.8 (25.2–30.0)	27.0 (25.4–30.4)	26.3 (24.7–29.3)	0.163
Dyslipidaemia	69 (93)	69 (64)	71 (29)	0.825
Arterial hypertension	80 (107)	81 (75)	78 (32)	0.730
Diabetes mellitus	27 (36)	24 (22)	34 (14)	0.207
Clinical presentation				
Typical angina	24 (32)	22 (20)	29 (12)	0.331
Atypical angina	27 (36)	25 (23)	32 (13)	0.401
Nonspecific chest pain	20 (27)	22 (20)	17 (7)	0.556
No chest pain	29 (39)	32 (30)	22 (9)	0.226
Positive stress test				
Electrocardiography	7 (9)	6 (6)	7 (3)	0.494
Echocardiography	14 (19)	11 (10)	22 (9)	0.087
Magnetic resonance perfusion imaging	17 (23)	16 (15)	20 (8)	0.632
Single-photon emission computed tomography	36 (48)	39 (36)	29 (12)	0.294
Pretest CAD status				
Prevalence of CAD				
Suspected or no obstructive disease	29 (39)	27 (25)	34 (14)	0.394
One-vessel disease	31 (42)	31 (29)	32 (13)	0.952
Two-vessel disease	22 (29)	24 (22)	17 (7)	0.394
Three-vessel disease	16 (22)	16 (15)	17 (7)	0.892
Four-vessel disease	1 (2)	2 (2)	0 (0)	0.999
Stress CTP parameters				
Contrast medium dose (ml) ^a^	60 (60–70)	60 (60–70)	60 (60–70)	0.130
Heart rate (bpm) ^a^	66.4 (61.5–74.5)	70.7 (64.9–76.4)	58.0 (53.3–64.1)	0.001
2-segment MSR	63 (84)	90 (84)	0 (0)	< 0.001
3-segment MSR	7 (9)	10 (9)	0 (0)	0.057
Per-segment temporal reconstruction window (ms) ^a^	129.7 (102.8–175.0)	115.3 (96.8–132.5)	175 (175–175)	< 0.001

Data are percentages with numbers of patients in parentheses, unless otherwise stated. ^a^Data are medians with interquartile ranges in parentheses (data not normally distributed). ^b^Calculated as $\frac{\mathrm{weight}\ \mathrm{in}\ \mathrm{kg}}{{\left(\mathrm{height}\ \mathrm{in}\ \mathrm{m}\right)}^2}$. ^c^Since a heart rate ≥ 65 bpm was the predefined cutoff for multisegment acquisition in the primary studies, the population for comparing the diagnostic performance of MSR and HSR of myocardial CTP consisted of 93 of 134 patients with multisegment acquisition of stress CTP allowing both MSR and HSR. The remaining 41 of 134 patients had single-segment acquisition due to heart rates < 65 bpm, and only HSR was possible. These patients served as a control group for interindividual radiation dose comparison (Fig. 1). *bpm* Beats per minute, *CAD* Coronary artery disease, *CTP* Computed tomography perfusion, *HSR* Halfscan reconstruction, *MSR* Multisegment reconstruction

### Diagnostic performance of MSR and HSR of myocardial CTP

Scan timing of static CTP was optimal to diagnose perfusion defects (Supplementary Table S2) [27]. Overall agreement between the two readers in identifying myocardial segments positive for perfusion defects was almost perfect in both per-patient (Cohen *κ* 0.94, raw agreement 96.9%) and per-territory analysis (0.97, 97.1%, respectively). Figure 3 shows a representative patient example juxtaposing stress CTP reconstructions (MSR and HSR) and the corresponding ICA images. We directly compare the results of MSR and HSR of myocardial CTP in the per-patient and per-territory analysis in relation to quantitative ICA in Supplementary Table S3. Additionally, direct comparison of the results of MSR and HSR of myocardial CTP by patient subgroup is presented in Supplementary Table S4 (per-patient) and in Supplementary Table S5 (per-territory). As one of the 93 patients we compared had no right coronary artery and 14 patients had a ramus intermedius, the 93 patients had a total of 292 myocardial artery territories that we analysed (left anterior descending artery territory, *n* = 93; left circumflex artery territory, *n* = 93; right coronary artery territory, *n* = 92; ramus intermedius territory, *n* = 14).

**Fig. 4:**
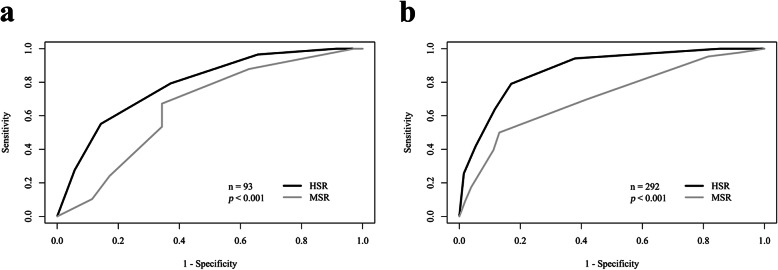
All patients: receiver operating characteristic areas under the curve for comparison of MSR and HSR of myocardial competed tomography perfusion in per-patient (**a**) and per-territory analysis (**b**) using 50% diameter stenosis detected on quantitative coronary angiography as a reference standard. In all 93 patients, the area under the curve of MSR was inferior to that of HSR for both levels of analysis (*p* < 0.001). *HSR* Halfscan reconstruction, *MSR* Multisegment reconstruction

The AUC of MSR was inferior to that of HSR on the per-patient level (93 patients; MSR 0.65 [95% confidence interval lower, upper 0.53, 0.78]; HSR 0.79 [0.69, 0.88]; *p* = 0.011) using 50% vessel stenosis detected in quantitative ICA as reference (Table 3, Fig. 4). Results of subgroup analysis including the diagnostic performance of MSR and HSR in patients with known CAD (68 of 93 patients), suspected CAD (25 of 93 patients), high heart rates ≥ 75 bpm (30 of 93 patients), and low heart rates (63 of 93 patients) using 50% vessel stenosis detected in ICA as reference are presented in Table 4 (per-patient) and Table 5 (per-territory). Per-patient AUC of MSR and HSR was similar in patients with known CAD (MSR 0.62 [0.45, 0.79]; HSR 0.72 [0.57, 0.86]; *p* = 0.157) and in patients with suspected CAD (MSR 0.80 [0.63, 0.97]; HSR 0.89 [0.77, 1.00]; *p* = 0.243) (Table 4). In addition, per-patient AUC of MSR and HSR was similar in patients with high heart rates (MSR 0.54 [0.27, 0.81]; HSR 0.55 [0.33, 0.77]; *p* = 0.611) whereas per-patient AUC of MSR was found to be inferior to that of HSR in patients with low heart rates (MSR 0.70 [0.57, 0.83]; HSR 0.87 [0.78, 0.95]; *p* = 0.007) (Table 4).

**Table 3:** All patients: diagnostic performance of MSR and HSR of myocardial CTP

	All 93 patients					
	Per-patient level			Per-territory level		
Reconstruction/performance	HSR	MSR	* p*-value	HSR	MSR	*p*-value
Area under the curve^a^	0.79 [0.69, 0.88]	0.65 [0.53, 0.78]	0.011	0.87 [0.83, 0.92]	0.71 [0.65, 0.78]	< 0.001
Sensitivity	88 (51/58) [77, 95]	67 (39/58) [54, 79]	0.001	79 (68/86) [69, 87]	50 (43/86) [39, 61]	< 0.001
Specificity	49 (17/35) [31, 66]	66 (23/35) [48, 81]	0.114	83 (171/206) [77, 88]	87 (179/206) [82, 91]	0.118
Positive predictive value	74 (51/69) [62, 84]	76 (39/51) [63, 87]	0.541	66 (68/103) [56, 75]	61 (43/70) [49, 73]	0.263
Negative predictive value	71 (17/24) [49, 87]	55 (23/42) [39, 70]	0.027	90 (171/189) [85, 94]	81 (179/222) [75, 86]	< 0.001

Reference: ≥ 50% diameter vessel stenosis detected in quantitative invasive coronary angiography. Data are the results of consensus reading of two readers. Unless otherwise stated, data are percentages, data in parentheses are raw data, and data in brackets are 95% confidence intervals. The 95% confidence intervals were estimated for unclustered data (see reference [31]). ^a^Data are the area under the curve and data in brackets are 95% confidence intervals. *CTP* Computed tomography perfusion, *HSR* Halfscan reconstruction, *MSR* Multisegment reconstruction

**Table 4:** Patient subgroups in per-patient level analysis: diagnostic performance of MSR and HSR of myocardial CTP

	Patients with known CAD			Patients with suspected CAD			Patients with high heart rates ≥ 75 bpm			Patients with low heart rates < 75 bpm		
Reconstruction/performance	HSR	MSR	*p*-value	HSR	MSR	*p*-value	HSR	MSR	*p*-value	HSR	MSR	*p*-value
Area under the curve^a^	0.72 [0.57, 0.86]	0.62 [0.45, 0.79]	0.157	0.89 [0.77, 1.00]	0.80 [0.63, 0.97]	0.243	0.55 [0.33, 0.77]	0.54 [0.27, 0.81]	0.611	0.87 [0.78, 0.95]	0.70 [0.57, 0.83]	0.007
Sensitivity	86 (43/50) [73, 94]	62 (31/50) [47, 75]	0.001	100 (8/8) [63, 100]	100 (8/8) [63, 100]	1.000	90 (19/21) [70, 99]	86 (18/21) [64, 97]	0.999	86 (32/37) [71, 95]	57 (21/37) [39, 73]	0.003
Specificity	39 (7/18) [17, 64]	67 (12/18) [41, 87]	0.131	59 (10/17) [33, 82]	65 (11/17) [38, 86]	0.999	11 (1/9) [0, 48]	33 (3/9) [7, 70]	0.480	62 (16/26) [41, 80]	77 (20/26) [56, 91]	0.289
Positive predictive value	80 (43/54) [66, 89]	84 (31/37) [68, 94]	0.387	53 (8/15) [27, 79]	57 (8/14) [29, 82]	0.563	70 (19/27) [50, 86]	75 (18/24) [53, 90]	0.254	76 (32/42) [61, 88]	78 (21/27) [58, 91]	0.814
Negative predictive value	50 (7/14) [23, 77]	39 (12/31) [22, 58]	0.282	100 (10/10) [69, 100]	100 (11/11) [72, 100]	0.999	33 (1/3) [1, 91]	50 (3/6) [12, 88]	0.417	76 (16/21) [53, 92]	56 (20/36) [38, 72]	0.009

Reference: ≥ 50% diameter vessel stenosis detected in quantitative invasive coronary angiography. Data are the results of consensus reading of two readers. Unless otherwise stated, data are percentages, data in parentheses are raw data, and data in brackets are 95% confidence intervals. The 95% confidence intervals were estimated for unclustered data (see reference [31]). ^a^Data are the area under the curve and data in brackets are 95% confidence intervals. *bpm* Beats per minute, *CAD* Coronary artery disease, *CTP* Computed tomography perfusion, *HSR* Halfscan reconstruction, *MSR* Multisegment reconstruction

**Table 5:** Patient subgroups in per-territory level analysis: diagnostic performance of MSR and HSR of myocardial CTP

	Patients with known CAD			Patients with suspected CAD			Patients with high heart rates ≥ 75 bpm			Patients with low heart rates < 75 bpm		
Reconstruction/performance	HSR	MSR	*p*-value	HSR	MSR	*p*-value	HSR	MSR	*p*-value	HSR	MSR	*p*-value
Area under the curve^a^	0.86 [0.81, 0.91]	0.72 [0.65, 0.79]	< 0.001	0.93 [0.87, 0.99]	0.71 [0.55, 0.87]	0.002	0.89 [0.83, 0.95]	0.74 [0.63, 0.85]	< 0.001	0.87 [0.82, 0.92]	0.70 [0.62, 0.78]	< 0.001
Sensitivity	78 (56/72) [66, 87]	49 (35/72) [37, 61]	< 0.001	86 (12/14) [57, 98]	57 (8/14) [29, 82]	0.134	90 (27/30) [73, 98]	60 (18/30) [41, 77]	0.008	73 (41/56) [60, 84]	45 (25/56) [31, 59]	< 0.001
Specificity	83 (119/143) [76, 89]	90 (128/143) [83, 94]	0.039	83 (52/63) [71, 91]	81 (51/63) [69, 90]	0.999	78 (49/63) [66, 87]	83 (52/63) [71, 91]	0.371	85 (122/143) [78, 91]	89 (127/143) [82, 93]	0.302
Positive predictive value	70 (56/80) [59, 80]	70 (35/50) [55, 82]	0.999	52 (12/23) [31, 73]	40 (8/20) [19, 62]	0.078	66 (27/41) [49, 80]	62 (18/29) [42, 79]	0.475	66 (41/62) [53, 78]	61 (25/41) [45, 76]	0.381
Negative predictive value	88 (119/135) [81, 93]	78 (128/165) [70, 84]	< 0.001	96 (52/54) [87, 100]	89 (51/57) [78, 96]	0.037	94 (49/52) [84, 99]	81 (52/64) [70, 90]	0.002	89 (122/137) [83, 94]	80 (127/158) [73, 86]	< 0.001

Reference: ≥ 50% diameter vessel stenosis detected in quantitative invasive coronary angiography. Data are the results of consensus reading of two readers. Unless otherwise stated, data are percentages, data in parentheses are raw data, and data in brackets are 95% confidence intervals. The 95% confidence intervals were estimated for unclustered data (see reference [31]). ^a^Data are the area under the curve and data in brackets are 95% confidence intervals. *bpm* Beats per minute, *CAD* Coronary artery disease, *CTP* Computed tomography perfusion, *HSR* Halfscan reconstruction, *MSR* Multisegment reconstruction

For all patients, diagnostic performance results of MSR and HSR of myocardial CTP using 70% vessel stenosis as reference are presented in Supplementary Table S6. Regarding subgroup analysis, the results for diagnostic performance of MSR and HSR of myocardial CTP using 70% vessel stenosis as reference are presented in Supplementary Table S7 (per-patient) and in Supplementary Table S8 (per-territory). In brief, per-patient analysis revealed the AUC of MSR and HSR of myocardial CTP to be similar (*p* > 0.050) in all patients and in each patient subgroup using 70% vessel stenosis detected in quantitative ICA as reference.

### Radiation dose

**Fig. 5:**
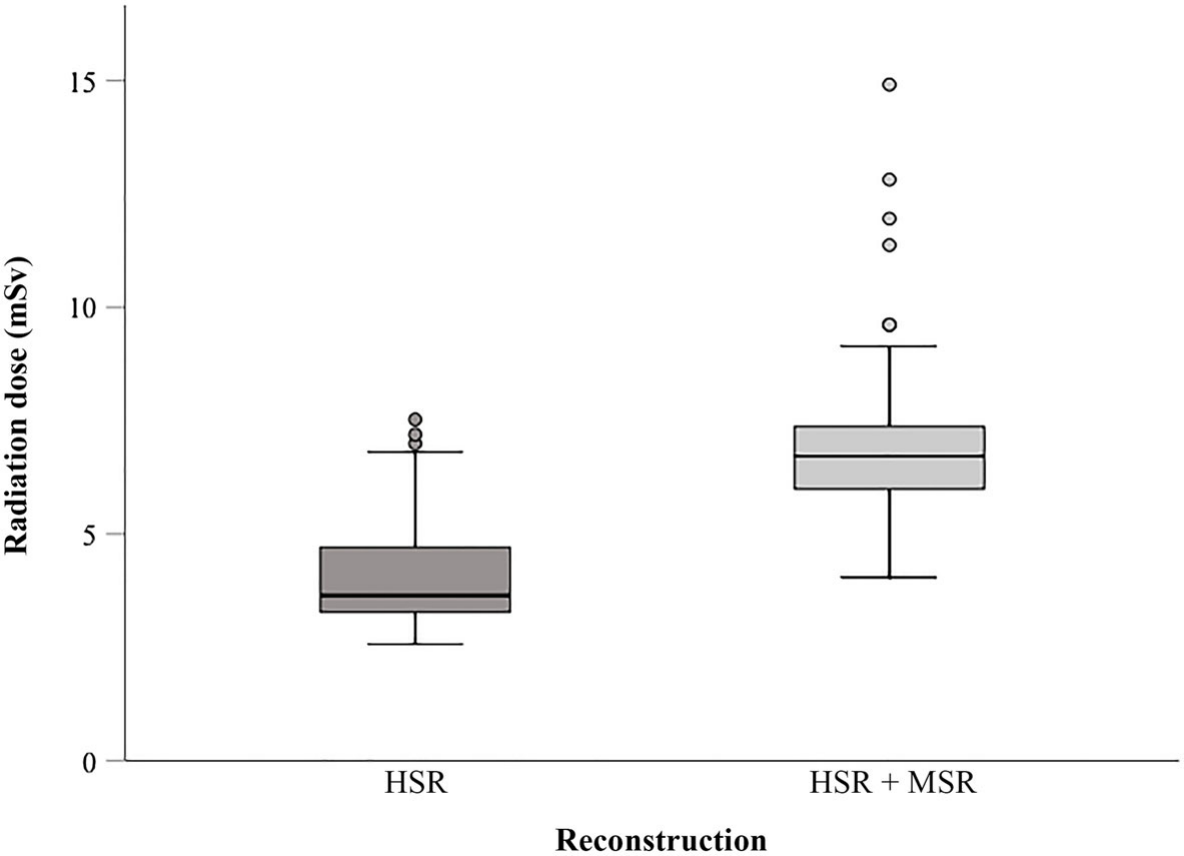
Boxplots showing estimated radiation dose in mSv of stress computed tomography perfusion in patients with multisegment acquisition, which allowed both MSR and HSR (93 patients), and in patients with HSR only (41 patients). Boundaries of boxes represent the lower and upper quartiles and horizontal lines in boxes the medians. Outliers are depicted as individual open circles. In the HSR-only group, MSR was not possible in 9 patients despite multisegment acquisition of two segments. Mean radiation dose of patients with both MSR and HSR was higher than that of patients with HSR (*p* < 0.001). *HSR* Halfscan reconstruction, *MSR* Multisegment reconstruction

Median estimated radiation dose of stress imaging was 6.67 mSv (interquartile range 5.98–7.36) in patients in whom both MSR and HSR was possible (93 of 134) and 3.64 mSv (3.27–4.71) in patients with HSR only (41 of 134; *p* < 0.001), resulting in approximately 45% higher radiation doses for MSR compared to HSR (Fig. 5). Considering rest-stress CTP, median estimated radiation dose was 10.53 mSv (interquartile range 9.11–12.11) in patients in whom both MSR and HSR was possible in stress CTP and 7.26 mSv (6.38–9.23) in patients with HSR only (*p* < 0.001).

## Discussion

We investigated whether MSR of myocardial CTP imaging can improve detection of obstructive CAD compared to HSR. The most important findings of our study are as follows: first, overall per-patient diagnostic performance of MSR was inferior to that of HSR; second, MSR also did not improve the AUC in any patient subgroup analysed (known/suspected CAD and high/low heart rate); and third, the radiation dose of MSR was higher than that of HSR.

The poorer diagnostic performance of MSR compared with HSR might in part be explained by a loss of partial resolution [8–11]. As MSR uses partial scanning data from several segments to reconstruct one volume, images can be blurred through image offsetting and averaging across the whole temporal reconstruction window of 230.6 ms [8–11]. This may have resulted in a higher number of false-negative results of MSR and consequently a lower sensitivity of MSR compared with HSR. The similarly low specificity of both reconstructions might be attributable to beam hardening artefacts leading to false positive results [38]. Additionally, non-diagnostic myocardial segments were classified as positive and included in our analysis, which possibly further lowered specificity [25] and may have led to higher overall agreement between the two readers. Conversely, older prospective research revealed higher diagnostic performance of MSR compared to HSR in assessing coronary arteries for CAD [7] whereas there was no difference in diagnostic performance between both reconstruction methods in the evaluation of global myocardial function despite using an older 16-row scanner [13]. The region of interest in CTP (myocardial perfusion defect) is larger than in CTA (coronary artery stenosis) and therefore may be accurately assessed by HSR despite the longer temporal reconstruction window of 175 ms. Consequently, implications of the results reported by the few older studies available on coronary CTA may not automatically be transferable to myocardial CTP. Investigating patients on a 64-row CT scanner, a later CTA study shows that shortening the temporal reconstruction window to 165 ms in HSR allows reliable detection of obstructive CAD by both MSR and HSR and that MSR cannot improve diagnostic performance compared to HSR [8]. This result suggests that HSR may benefit more than MSR from faster scanners with more detector rows in terms of diagnostic performance when performing CTA to diagnose CAD. The larger number of detector rows now available enables whole-heart coverage (no pitch), preventing stair-step artefacts, which compromised both HSR and MSR in older studies, while HSR was additionally limited by more motion artefacts due to the longer temporal reconstruction window compared to MSR [12]. Our findings obtained using 320-row CT datasets suggest MSR to be inferior to HSR for detection of myocardial perfusion defects. However, to our knowledge, we are the first to compare the diagnostic performance of MSR and HSR in myocardial CTP. The fact that MSR was so far investigated only for CTA and myocardial function in times when CT scanners were much slower and did not offer whole-heart coverage makes it difficult to determine which factors contributed most to our different results for CTP *versus* CTA—either the technical advances in CT scanner technology and protocols or the fact that we now diagnose CAD by evaluating the myocardium instead of the coronary arteries.

Our subgroup analysis showed diagnostic performance of MSR and HSR to be similar in the two patient groups with known and suspected CAD. As no power analysis was conducted for this retrospective substudy, differences between MSR and HSR may be less apparent due to a small number of patients especially in the subgroup with suspected CAD (25 patients). However, the results in these subgroups support our overall finding that MSR does not improve the diagnostic performance on any level analysis compared to HSR. For patients with high heart rates, the few available older prospective CTA studies showed the diagnostic performance of MSR in detecting obstructive CAD to be higher [7] or similar [8] compared with HSR [8]. For myocardial CTP imaging, our findings show that MSR and HSR have similar diagnostic performance with high sensitivities in patients with heart rates > 75 bpm, suggesting that the temporal reconstruction window of HSR may be short enough and MSR therefore does not offer additional benefits for diagnosing CAD using CTP when patients are examined in a later-generation CT scanner. However, the AUC of both MSR and HSR in this patient subgroup is smaller, which may be driven by a low specificity possibly attributable to an increase in motion artefacts with rising heart rates [39] that may compromise both reconstruction techniques.

A major concern of myocardial CTP with MSR is that acquiring more segments increases radiation exposure [11, 40–42]. Our estimated radiation dose saving of 45% of single-segment instead of multisegment acquisition is comparable with dose savings reported in the literature (21.6% [41] to 52.0% [40]). Thus, our findings in CTP might be generalizable for studies using first generation 320-row CT scanners. In contrast, latest scanner generations using static and even dynamic CTP protocols achieve even lower radiation doses than in our study [19, 43] as we analysed raw data acquired 10 years ago. Furthermore, use of dose reduction strategies such as electrocardiogram-gated tube current modulation may contribute to further dose reduction [44]. However, the tendency that radiation dose is higher in static CTP when using a multisegment acquisition protocol followed by MSR instead of a single-segment acquisition followed by HSR may be the same even for examinations performed using the latest-generation CT scanners and protocols.

Our study is limited by the use of quantitative ICA as predefined reference standard rather than invasive fractional flow reserve. This may have influenced the reference outcome in any way. To consider functional relevance, we additionally included ≥ 70% diameter stenosis diameter as secondary reference standard as this diameter cut-off was associated with a perfusion defect in single-photon emission tomography in a subgroup of the CORE320 cohort [16]. Using this secondary reference, we still found overall AUC of MSR and HSR to be similar, indicating that MSR cannot improve the diagnostic performance of CTP compared to HSR even in patients with high-grade stenosis. Another limitation is that subgroups included only small numbers of patients as no additional power analysis was conducted for this retrospective study. However, in no patient subgroup did MSR improve diagnostic performance, but MSR showed similar or smaller AUCs compared to HSR. Furthermore, as the primary studies were conducted in 2011, gantry rotation times were longer than available today. However, the overall inferiority of MSR to HSR might even be more significant in faster CT scanners due to the even shorter temporal reconstruction window of HSR that is not affected by the previously suggested limitations of MSR.

In conclusion, our results showed that MSR does not improve diagnostic performance of myocardial CTP imaging for detection of obstructive CAD while increasing radiation doses compared with HSR.

## Supplementary Information

**Additional file 1: Supplementary Table 1.** Primary objectives as well as inclusion and exclusion criteria of the primary studies. **Supplementary Table 2.** Scan timing of static myocardial CT perfusion during arterial contrast medium first pass. **Supplementary Table 3.** All patients: direct comparison of the results of MSR and HSR of myocardial CTP. **Supplementary Table 4.** Patient subgroups in per-patient level analysis: direct comparison of the results of MSR and HSR of myocardial CTP. **Supplementary Table 5.** Patient subgroups in per-territory level analysis: direct comparison of the results of MSR and HSR of myocardial CTP. **Supplementary Table 6.** All patients using ≥ 70% stenosis as reference: diagnostic performance of MSR and HSR of myocardial CTP. **Supplementary Table 7.** Patient subgroups in per-patient level analysis using ≥ 70% stenosis as reference: diagnostic performance of MSR and HSR of myocardial CTP. **Supplementary Table 8.** Patient subgroups in per-territory level analysis using ≥ 70% stenosis as reference: diagnostic performance of MSR and HSR of myocardial CTP.

## Data Availability

The datasets used and/or analysed during the current study are available from the corresponding author on reasonable request. The dataset supporting the conclusions of this article is included within the article.

## Abbreviations

AUC: Area under the curve
bpm: Beats per minute
CAD: Coronary artery disease
CTA: Computed tomography angiography
CTP: Computed tomography perfusion
HSR: Halfscan reconstruction
ICA: Invasive coronary angiography
MSR: Multisegment reconstruction

## References

[1] Fihn SD, Gardin JM, Abrams J (2012). 2012 ACCF/AHA/ACP/AATS/PCNA/SCAI/STS Guideline for the diagnosis and management of patients with stable ischemic heart disease: a report of the American College of Cardiology Foundation/American Heart Association Task Force on Practice Guidelines, and the American College of Physicians, American Association for Thoracic Surgery, Preventive Cardiovascular Nurses Association, Society for Cardiovascular Angiography and Interventions, and Society of Thoracic Surgeons. J Am Coll Cardiol.

[2] Montalescot G, Sechtem U, Achenbach S (2013). 2013 ESC guidelines on the management of stable coronary artery disease: the Task Force on the management of stable coronary artery disease of the European Society of Cardiology. Eur Heart J.

[3] George RT, Mehra VC, Chen MY, et al. (2014) Myocardial CT perfusion imaging and SPECT for the diagnosis of coronary artery disease: a head-to-head comparison from the CORE320 multicenter diagnostic performance study. Radiology 272:407–416 10.1148/radiol.14140806 10.1148/radiol.14140806 PMC4263655 24865312

[4] Rief M, Chen MY, Vavere AL, et al. (2018) Coronary artery disease: analysis of diagnostic performance of CT perfusion and MR perfusion imaging in comparison with quantitative coronary angiography and SPECT-multicenter prospective trial. Radiology 286:461–470 10.1148/radiol.2017162447 10.1148/radiol.2017162447 PMC5790301 28956734

[5] Sorgaard MH, Kofoed KF, Linde JJ (2016). Diagnostic accuracy of static CT perfusion for the detection of myocardial ischemia. A systematic review and meta-analysis. J Cardiovasc Comput Tomogr.

[6] Celeng C, Leiner T, Maurovich-Horvat P, et al. (2019) Anatomical and functional computed tomography for diagnosing hemodynamically significant coronary artery disease: a meta-analysis. JACC Cardiovasc Imaging 12:1316–1325 10.1016/j.jcmg.2018.07.022 10.1016/j.jcmg.2018.07.022 30219398

[7] Dewey M, Teige F, Laule M, Hamm B (2007). Influence of heart rate on diagnostic accuracy and image quality of 16-slice CT coronary angiography: comparison of multisegment and halfscan reconstruction approaches. Eur Radiol.

[8] Herzog C, Nguyen SA, Savino G, et al. (2007) Does two-segment image reconstruction at 64-section CT coronary angiography improve image quality and diagnostic accuracy? Radiology 244:121–129 10.1148/radiol.2441060004 10.1148/radiol.2441060004 17495177

[9] Taguchi K, Anno H (2000). High temporal resolution for multislice helical computed tomography. Med Phys.

[10] Flohr T, Ohnesorge B (2001). Heart rate adaptive optimization of spatial and temporal resolution for electrocardiogram-gated multislice spiral CT of the heart. J Comput Assist Tomogr.

[11] Dewey M, Laule M, Krug L, et al. (2004) Multisegment and halfscan reconstruction of 16-slice computed tomography for detection of coronary artery stenoses. Invest Radiol 39:223–229 10.1097/01.rli.0000115201.27096.6e 10.1097/01.rli.0000115201.27096.6e 15021326

[12] Schnapauff D, Teige F, Hamm B, Dewey M (2009). Comparison between the image quality of multisegment and halfscan reconstructions of non-invasive CT coronary angiography. Br J Radiol.

[13] Dewey M, Muller M, Teige F (2006). Multisegment and halfscan reconstruction of 16-slice computed tomography for assessment of regional and global left ventricular myocardial function. Invest Radiol.

[14] Sørgaard MH, Linde JJ, Kühl JT, et al. (2018) Value of myocardial perfusion assessment with coronary computed tomography angiography in patients with recent acute-onset chest pain. JACC Cardiovasc Imaging 11:1611–1621 10.1016/j.jcmg.2017.09.022 10.1016/j.jcmg.2017.09.022 29248654

[15] Ko BS, Linde JJ, Ihdayhid AR, et al. (2019) Non-invasive CT-derived fractional flow reserve and static rest and stress CT myocardial perfusion imaging for detection of haemodynamically significant coronary stenosis. Int J Cardiovasc Imaging 35:2103–2112 10.1007/s10554-019-01658-x 10.1007/s10554-019-01658-x PMC6805817 31273632

[16] Bakhshi H, Meyghani Z, Kishi S, et al. (2019) Comparative effectiveness of CT-derived atherosclerotic plaque metrics for predicting myocardial ischemia. JACC Cardiovasc Imaging 12:1367–1376 10.1016/j.jcmg.2018.05.019 10.1016/j.jcmg.2018.05.019 30031705

[17] Pontone G, Baggiano A, Andreini D, et al. (2019) Stress computed tomography perfusion versus fractional flow reserve CT derived in suspected coronary artery disease: the PERFECTION study. JACC Cardiovasc Imaging 12:1487–1497 10.1016/j.jcmg.2018.08.023 10.1016/j.jcmg.2018.08.023 30343073

[18] Andreini D, Mushtaq S, Pontone G, et al. (2020) CT perfusion versus coronary CT angiography in patients with suspected in-stent restenosis or CAD progression. JACC Cardiovasc Imaging 13:732–742 10.1016/j.jcmg.2019.05.031 10.1016/j.jcmg.2019.05.031 31422127

[19] Bechsgaard DF, Gustafsson I, Michelsen MM, et al. (2020) Evaluation of computed tomography myocardial perfusion in women with angina and no obstructive coronary artery disease. Int J Cardiovasc Imaging 36:367–382 10.1007/s10554-019-01723-5 10.1007/s10554-019-01723-5 31676944

[20] Vavere AL, Simon GG, George RT, et al. (2011) Diagnostic performance of combined noninvasive coronary angiography and myocardial perfusion imaging using 320 row detector computed tomography: design and implementation of the CORE320 multicenter, multinational diagnostic study. J Cardiovasc Comput Tomogr 5:370–381 10.1016/j.jcct.2011.11.001 10.1016/j.jcct.2011.11.001 PMC3828643 22146496

[21] Rochitte CE, George RT, Chen MY, et al. (2014) Computed tomography angiography and perfusion to assess coronary artery stenosis causing perfusion defects by single photon emission computed tomography: the CORE320 study. Eur Heart J 35:1120–1130 10.1093/eurheartj/eht488 10.1093/eurheartj/eht488 PMC6693293 24255127

[22] Rief M, Zimmermann E, Stenzel F, et al. (2013) Computed tomography angiography and myocardial computed tomography perfusion in patients with coronary stents: prospective intraindividual comparison with conventional coronary angiography. J Am Coll Cardiol 62:1476–1485 10.1016/j.jacc.2013.03.088 10.1016/j.jacc.2013.03.088 23792193

[23] Tomizawa N, Yamamoto K, Akahane M, Torigoe R, Kiryu S, Ohtomo K (2013). The feasibility of halfcycle reconstruction in high heart rates in coronary CT angiography using 320-row CT. Int J Cardiovasc Imaging.

[24] George RT, Arbab-Zadeh A, Cerci RJ, et al. (2011) Diagnostic performance of combined noninvasive coronary angiography and myocardial perfusion imaging using 320-MDCT: the CT angiography and perfusion methods of the CORE320 multicenter multinational diagnostic study. AJR Am J Roentgenol 197:829–837 10.2214/ajr.10.5689 10.2214/AJR.10.5689 PMC3302727 21940569

[25] Schuetz GM, Schlattmann P, Dewey M (2012). Use of 3 × 2 tables with an intention to diagnose approach to assess clinical performance of diagnostic tests: meta-analytical evaluation of coronary CT angiography studies. BMJ.

[26] Cerqueira MD, Weissman NJ, Dilsizian V, et al. (2002) Standardized myocardial segmentation and nomenclature for tomographic imaging of the heart. A statement for healthcare professionals from the Cardiac Imaging Committee of the Council on Clinical Cardiology of the American Heart Association. Circulation 105:539–542 10.1161/hc0402.102975 10.1161/hc0402.102975 11815441

[27] Bischoff B, Bamberg F, Marcus R, et al. (2013) Optimal timing for first-pass stress CT myocardial perfusion imaging. Int J Cardiovasc Imaging 29:435–442 10.1007/s10554-012-0080-y 10.1007/s10554-012-0080-y 22714549

[28] George RT, Jerosch-Herold M, Silva C, et al. (2007) Quantification of myocardial perfusion using dynamic 64-detector computed tomography. Invest Radiol 42:815–822 10.1097/RLI.0b013e318124a884 10.1097/RLI.0b013e318124a884 18007153

[29] George RT, Silva C, Cordeiro MA (2006). Multidetector computed tomography myocardial perfusion imaging during adenosine stress. J Am Coll Cardiol.

[30] DeLong ER, DeLong DM, Clarke-Pearson DL (1988). Comparing the areas under two or more correlated receiver operating characteristic curves: a nonparametric approach. Biometrics.

[31] Newcombe R, Altman D, Altman D, Machin D, Bryant T, Gardner M (2000). Proportions and their differences. Statistics with confidence.

[32] Leisenring W, Alonzo T, Pepe MS (2000). Comparisons of predictive values of binary medical diagnostic tests for paired designs. Biometrics.

[33] McHugh ML (2012). Interrater reliability: the kappa statistic. Biochem Med (Zagreb).

[34] R Core Team (2017) R: A language and environment for statistical computing. R Foundation for Statistical Computing, Vienna, Austria. url:https://www.r-project.org/

[35] Robin X, Turck N, Hainard A, et al. (2011) pROC: an open-source package for R and S+ to analyze and compare ROC curves. BMC Bioinformatics 12:77 10.1186/1471-2105-12-77 10.1186/1471-2105-12-77 PMC3068975 21414208

[36] Stock C, Hielscher T (2014) DTComPair: comparison of binary diagnostic tests in a paired study design. R package version 1.0.3. url:http://cran.r-project.org/package = DTComPair

[37] Stevenson M (2017) epiR: Tools for the analysis of epidemiological data. R package version 0.9-91. url:https://cran.r-project.org/package = epiR

[38] Carrascosa PM, Cury RC, Deviggiano A, et al. (2015) Comparison of myocardial perfusion evaluation with single versus dual-energy CT and effect of beam-hardening artifacts. Acad Radiol 22:591–599 10.1016/j.acra.2014.12.019 10.1016/j.acra.2014.12.019 25680523

[39] Steveson C, Schuijf JD, Vavere AL, et al. (2017) The effect of heart rate on exposure window and best phase for stress perfusion computed tomography: lessons from the CORE320 study. J Comput Assist Tomogr 41:242–248 10.1097/rct.0000000000000514 10.1097/RCT.0000000000000514 28288480

[40] Lee AB, Nandurkar D, Schneider-Kolsky ME, et al. (2011) Coronary image quality of 320-MDCT in patients with heart rates above 65 beats per minute: preliminary experience. AJR Am J Roentgenol 196:W729–W735 10.2214/ajr.10.5252 10.2214/AJR.10.5252 21606261

[41] Huang W, Xu Y, Lu D, Shi Y, Lu G (2015). Single- versus multi-phase acquisition protocol for prospective-triggered sequential dual-source CT coronary angiography: comparison of image quality and radiation dose. Clin Imaging.

[42] Pelgrim GJ, Dorrius M, Xie X, et al. (2015) The dream of a one-stop-shop: Meta-analysis on myocardial perfusion CT. Eur J Radiol 84:2411–2420 10.1016/j.ejrad.2014.12.032 10.1016/j.ejrad.2014.12.032 25636388

[43] Yu M, Shen C, Dai X, et al. (2020) Clinical outcomes of dynamic computed tomography myocardial perfusion imaging combined with coronary computed tomography angiography versus coronary computed tomography angiography-guided strategy. Circ Cardiovasc Imaging 13:e009775 10.1161/circimaging.119.009775 10.1161/CIRCIMAGING.119.009775 31910669

[44] He G, Liu X, Liu Y, Wang W, Ke Z (2015). Dose study of electrocardiogram automatic tube current modulation technology in prospective coronary computed tomography angiography scans of overweight patients. Exp Ther Med.

