# Supplementary material for: Myocardial CT perfusion imaging for the detection of obstructive coronary artery disease: multisegment reconstruction does not improve diagnostic performance
Source: Eur Radiol Exp. 2022 Jan 31;6:5. doi: 10.1186/s41747-021-00256-8 (PMC8804122; doi:10.1186/s41747-021-00256-8)
Supplement: Supplementary file 1 — Additional file 1: Supplementary Table 1. Primary objectives as well as inclusion and exclusion criteria of the primary studies. Supplementary Table 2. Scan timing of static myocardial CT perfusion during arterial contrast medium first pass. Supplementary Table 3. All patients: direct comparison of the results of MSR and HSR of myocardial CTP. Supplementary Table 4. Patient subgroups in per-patient level analysis: direct comparison of the results of MSR and HSR of myocardial CTP. Supplementary Table 5. Patient subgroups in per-territory level analysis: direct comparison of the results of MSR and HSR of myocardial CTP. Supplementary Table 6. All patients using ≥ 70% stenosis as reference: diagnostic performance of MSR and HSR of myocardial CTP. Supplementary Table 7. Patient subgroups in per-patient level analysis using ≥ 70% stenosis as reference: diagnostic performance of MSR and HSR of myocardial CTP. Supplementary Table 8. Patient subgroups in per-territory level analysis using ≥ 70% stenosis as reference: diagnostic performance of MSR and HSR of myocardial CTP. [file 41747_2021_256_MOESM1_ESM.docx]

# ELECTRONIC SUPPLEMENTARY MATERIAL

# Supplementary Table 1. Primary objectives as well as inclusion and exclusion criteria of the primary studies

| **Design** | **CORE320 [20]** |  |  | |  |  | **CARS-320 [22]** |
| --- | --- | --- | --- | --- | --- | --- | --- |
|  |  |  |  | |  |  |  |
| Objectives | To compare the diagnostic performance of the combination of CTA and myocardial CTP imaging with the combination of quantitative ICA and SPECT as first reference and quantitative ICA alone as second reference for the detection of obstructive CAD in patients with known or suspected CAD | | | | |  | To compare the diagnostic performance of coronary CTA alone with the combination of coronary CTA and myocardial CTP for the detection of in-stent restenosis in patients with coronary stents using quantitative ICA as reference |
|  |  |  | |  |  |  |  |
|  |  |  | |  |  |  |  |
| Inclusion criteria | Patient with known or suspected CAD and clinical indication for ICA | | |  |  |  | Patient with suspected coronary in-stent restenosis and clinical indication for ICA |
|  |  |  | |  |  |  |  |
|  |  |  | |  |  |  |  |
| Exclusion criteria | Coronary artery bypass graft or other cardiac surgery | | |  |  |  | Coronary artery bypass graft |
|  | Atrial fibrillation or uncontrolled tachyarrhythmia, > 1st degree artrioventricular block | | | | |  | No sinus rhythm, > 1st degree atrioventricular block |
|  | Sinus bradycardia < 40 bpm |  |  | |  |  |  |
|  | Unstable acute coronary syndrome or myocardial infarction | |  | |  |  | Unstable presentation |
|  | Serum creatinine > 1.5 mg/dl or calculated creatinine clearance of < 60 ml/min | | | |  |  | Serum creatinine > 2.0 mg/dl |
|  | History of contrast-medium-induced nephropathy | |  | |  |  |  |
|  | Body mass index > 40 |  |  | |  |  | Body weight > 300 kg |
|  | Systolic blood pressure < 90 mmHg, recent use of dipyridamole or methylxanthines | | | |  |  | Systolic hypotension, therapy with dipyridamole |
|  | Age < 45 or > 85 years |  |  | |  |  | Age < 40 years |
|  | Pregnancy |  |  | |  |  | Pregnancy |
|  | Patient not willing or unable to sign written informed consent | |  | |  |  | Guardianship at the time of the study |
|  | Coronary artery intervention within the last 6 months | |  | |  |  | Inability to hold breath for 10 seconds |
|  | Known allergy to iodinated contrast media |  |  | |  |  |  |
|  | Known or suspected intolerance or contraindication to beta-blockers | | | |  |  |  |
|  | Evidence of severe symptomatic heart failure (NYHA class III or IV) | | | |  |  |  |
|  | Known or suspected moderate or severe aortic stenosis | |  | |  |  |  |
|  | Presence of intracardiac devices within the imaging field of view | | | |  |  |  |
|  | Chronic obstructive pulmonary disease with use of inhaled broncodialators over the past year | | | | |  |  |
|  | History of multiple myeloma or previous organ transplantation | |  | |  |  |  |
|  | Clinical SPECT preformed by non-validated center within 60 days prior to screening | | | |  |  |  |
|  | Clinical SPECT in the period 6 to 3 months prior to screening | |  | |  |  |  |
|  | Dual isotope, thallium or sestamibi imaging studies including rest and stress studies with 2-day protocols | | | | |  |  |
|  | Patients with a history of high radiation exposure | |  | |  |  |  |
|  | Any other history or condition that the investigator judged to be a significant reason for exclusion | | | | |  |  |

*bpm* beats per minute, *CAD* coronary artery disease, *CTA* computed tomography angiography, *CTP* computed tomography perfusion, *ICA* invasive coronary angiography, *SPECT* single-photon emission computed tomography

# Supplementary Table 2. Scan timing of static myocardial CT perfusion during arterial contrast medium first pass

| **Location** | **CT attenuation (HU)^a^** | ***p*- value** |
| --- | --- | --- |
| Left ventricle (A) | 369.0 (89.3) | < 0.001 (A versus B)  < 0.001 (A versus C)  < 0.001 (A versus D)  0.129 (B versus C)  < 0.001 (B versus D)  < 0.001 (C versus D) |
| Ascending aorta (B) | 394.1 (83.8) |  |
| Proximal descending aorta (C) | 399.6 (85.9) |  |
| Distal descending aorta (D) | 353.2 (72.1) |  |

Patients were scanned with the peak of the contrast medium bolus in or having just left the ascending aorta - which is assumed to be the phase with optimal demarcation of perfusion defects in static myocardial CT perfusion [see reference 27]. a Data are means with standard deviations in parentheses (data normally distributed). *HU* Hounsfield units

**Supplementary Table 3.** All patients: direct comparison of the results of MSR and HSR of myocardial CTP

|  | **ICA finding** | | | | | | |
| --- | --- | --- | --- | --- | --- | --- | --- |
|  | **Per-patient level** | | |  | **Per-territory level** | | |
| **Reconstruction / finding** | *Positive* | *Negative* | *Total* |  | *Positive* | *Negative* | *Total* |
| HSR |  |  |  |  |  |  |  |
| *Positive* | 51 | 18 | 69 |  | 68 | 35 | 103 |
| *Negative* | 7 | 17 | 24 |  | 18 | 171 | 189 |
| *Total* | 58 | 35 | 93 |  | 86 | 206 | 292 |
| MSR |  |  |  |  |  |  |  |
| *Positive* | 39 | 12 | 51 |  | 43 | 27 | 70 |
| *Negative* | 19 | 23 | 42 |  | 43 | 179 | 222 |
| *Total* | 58 | 35 | 93 |  | 86 | 206 | 292 |

Reference: ≥ 50% diameter vessel stenosis detected in quantitative ICA. Data are the results of consensus reading of two readers. Myocardial perfusion defects in MSR and HSR were manually assigned to their culprit lesions detected in ICA using thin-slice rest CTP images. *CTP* computed tomography perfusion, *HSR* halfscan reconstruction, *ICA* invasive coronary angiography, *MSR* multisegment reconstruction

**Supplementary Table 4.** Patient subgroups in per-patient level analysis: direct comparison of the results of MSR and HSR of myocardial CTP

|  | **ICA finding** | | | | | | | | | | | | | | |  |
| --- | --- | --- | --- | --- | --- | --- | --- | --- | --- | --- | --- | --- | --- | --- | --- | --- |
|  | **Patients with  known CAD** | | |  | **Patients with  suspected CAD** | | |  | **Patients with  high heart rates  ≥ 75 bpm** | | |  | **Patients with  low heart rates  < 75 bpm** | | |  |
| **Reconstruction / finding** | *Positive* | *Negative* | *Total* |  | *Positive* | *Negative* | *Total* |  | *Positive* | *Negative* | *Total* |  | *Positive* | *Negative* | *Total* |  |
| HSR |  |  |  |  |  |  |  |  |  |  |  |  |  |  |  |  |
| *Positive* | 43 | 11 | 54 |  | 8 | 7 | 15 |  | 19 | 8 | 27 |  | 32 | 10 | 42 |  |
| *Negative* | 7 | 7 | 14 |  | 0 | 10 | 10 |  | 2 | 1 | 3 |  | 5 | 16 | 21 |  |
| *Total* | 50 | 18 | 68 |  | 8 | 17 | 25 |  | 21 | 9 | 30 |  | 37 | 26 | 63 |  |
| MSR |  |  |  |  |  |  |  |  |  |  |  |  |  |  |  |  |
| *Positive* | 31 | 6 | 37 |  | 8 | 6 | 14 |  | 18 | 6 | 24 |  | 21 | 6 | 27 |  |
| *Negative* | 19 | 12 | 31 |  | 0 | 11 | 11 |  | 3 | 3 | 6 |  | 16 | 20 | 36 |  |
| *Total* | 50 | 18 | 68 |  | 8 | 17 | 25 |  | 21 | 9 | 30 |  | 37 | 26 | 63 |  |

Reference: ≥ 50% diameter vessel stenosis detected in quantitative ICA. Data are the results of consensus reading of two readers. Myocardial perfusion defects in MSR and HSR were manually assigned to their culprit lesions detected in ICA using thin-slice rest CTP images. *bpm* beats per minute, *CAD* coronary artery disease, *CTP* computed tomography perfusion, *HSR* halfscan reconstruction, *ICA* invasive coronary angiography, *MSR* multisegment reconstruction

**Supplementary Table 5.** Patient subgroups in per-territory level analysis: direct comparison of the results of MSR and HSR of myocardial CTP

|  | **ICA finding** | | | | | | | | | | | | | | |  |  |
| --- | --- | --- | --- | --- | --- | --- | --- | --- | --- | --- | --- | --- | --- | --- | --- | --- | --- |
|  | **Patients with**  **known CAD** | | |  | **Patients with**  **suspected CAD** | | |  | **Patients with  high heart rates  ≥ 75 bpm** | | |  | **Patients with  low heart rates  < 75 bpm** | | | |  |
| **Reconstruction / finding** | *Positive* | *Negative* | *Total* |  | *Positive* | *Negative* | *Total* |  | *Positive* | *Negative* | *Total* |  | *Positive* | *Negative* | *Total* | |  |
| HSR |  |  |  |  |  |  |  |  |  |  |  |  |  |  |  | |  |
| *Positive* | 56 | 24 | 80 |  | 12 | 11 | 23 |  | 27 | 14 | 41 |  | 41 | 21 | 62 | |  |
| *Negative* | 16 | 119 | 135 |  | 2 | 52 | 54 |  | 3 | 49 | 52 |  | 15 | 122 | 137 | |  |
| *Total* | 72 | 143 | 215 |  | 14 | 63 | 77 |  | 30 | 63 | 93 |  | 56 | 143 | 199 | |  |
| MSR |  |  |  |  |  |  |  |  |  |  |  |  |  |  |  | |  |
| *Positive* | 35 | 15 | 50 |  | 8 | 12 | 20 |  | 18 | 11 | 29 |  | 25 | 16 | 41 | |  |
| *Negative* | 37 | 128 | 165 |  | 6 | 51 | 57 |  | 12 | 52 | 64 |  | 31 | 127 | 158 | |  |
| *Total* | 72 | 143 | 215 |  | 14 | 63 | 77 |  | 30 | 63 | 93 |  | 56 | 143 | 199 | |  |

Reference: ≥ 50% diameter vessel stenosis detected in quantitative ICA. Data are the results of consensus reading of two readers. Myocardial perfusion defects in MSR and HSR were manually assigned to their culprit lesions detected in ICA using thin-slice rest CTP images. *bpm* beats per minute, *CAD* coronary artery disease, *CTP* computed tomography perfusion, *HSR* halfscan reconstruction, *ICA* invasive coronary angiography, *MSR* multisegment reconstruction

# Supplementary Table 6. All patients using ≥ 70% stenosis as reference: diagnostic performance of MSR and HSR of myocardial CTP

|  |  | **All 93 patients** | | | | | |  | |  |
| --- | --- | --- | --- | --- | --- | --- | --- | --- | --- | --- |
|  |  | **Per-patient level** | | |  | **Per-territory level** | | |  | |
| **Reconstruction /**  **performance** |  | **HSR** | **MSR** | ***p*- value** |  | **HSR** | **MSR** | ***p*- value** |  | |
| Area under the curve^a^ |  | 0.70  [0.59, 0.81] | 0.67  [0.55, 0.78] | 0.503 |  | 0.85  [0.79, 0.91] | 0.70  [0.61, 0.80] | < 0.001 |  | |
| Sensitivity |  | 92 (24/26) [75, 99] | 73 (19/26) [52, 88] | 0.074 |  | 88 (28/32)  [71, 96] | 53 (17/32)  [35, 71] | 0.003 |  | |
| Specificity |  | 33 (22/67) [22, 45] | 52 (35/67) [40, 65] | 0.004 |  | 71 (185/260)  [65, 77] | 80 (207/260) [74, 84] | < 0.001 |  | |
| Positive predictive value |  | 35 (24/69) [24, 47] | 37 (19/51) [24, 52] | 0.483 |  | 27 (28/103)  [19, 37] | 24 (17/70)  [15, 36] | 0.384 |  | |
| Negative predictive value |  | 92 (22/24) [73, 99] | 83 (35/42) [69, 93] | 0.124 |  | 98 (185/189)  [95, 99] | 93 (207/222) [89, 96] | 0.001 |  | |

Reference: ≥ 70% diameter vessel stenosis detected in quantitative ICA. Data are the results of consensus reading of two readers. Unless otherwise stated, data are percentages, data in parentheses are raw data, and data in brackets are 95% confidence intervals. The 95% confidence intervals were estimated for unclustered data [see reference 31]. ^a^ Data are the area under the curve and data in brackets are 95% confidence intervals. *CTP* computed tomography perfusion, *HSR* halfscan reconstruction, *ICA* invasive coronary angiography, *MSR* multisegment reconstruction

**Supplementary Table 7.** Patient subgroups in per-patient level analysis using ≥ 70% stenosis as reference: diagnostic performance of MSR and HSR of myocardial CTP

|  | **Patients with known CAD** | | |  | **Patients with suspected CAD** | | |  | **Patients with high heart rates  ≥ 75 bpm** | | | |  | **Patients with low heart rates  < 75 bpm** | | |
| --- | --- | --- | --- | --- | --- | --- | --- | --- | --- | --- | --- | --- | --- | --- | --- | --- |
| **Reconstruction /**  **performance** | **HSR** | **MSR** | ***p*- value** |  | **HSR** | **MSR** | ***p*- value** |  | **HSR** | **MSR** | ***p*- value** | |  | **HSR** | **MSR** | ***p*- value** |
| Area under the curve^a^ | 0.63  [0.49, 0.77] | 0.63  [0.48, 0.77] | 0.975 |  | 0.89  [0.78, 1.00] | 0.78  [0.61, 0.96] | 0.114 |  | 0.60  [0.36, 0.84] | 0.62  [0.36, 0.89] | | 0.704 |  | 0.77  [0.64, 0.89] | 0.72  [0.58, 0.85] | 0.475 |
| Sensitivity | 90 (18/20) [68, 99] | 65 (13/20) [41, 85] | 0.074 |  | 100 (6/6) [54, 100] | 100 (6/6) [54, 100] | 1.000 |  | 100 (7/7) [59, 100] | 86 (6/7) [42, 100] | | 0.999 |  | 89 (17/19) [67, 99] | 68 (13/19) [43, 87] | 0.134 |
| Specificity | 25 (12/48) [14, 40] | 50 (24/48) [35/65] | 0.003 |  | 53 (10/19) [29, 76] | 58 (11/19) [33, 80] | 0.999 |  | 13 (3/23) [3, 34] | 22 (5/23) [7, 44] | | 0.480 |  | 43 (19/44) [28, 59] | 68 (30/44) [52, 81] | 0.010 |
| Positive predictive value | 33 (18/54) [21, 47] | 35 (13/37) [20, 52] | 0.683 |  | 40 (6/15) [16, 68] | 43 (6/14) [18, 71] | 0.565 |  | 26 (7/27) [11, 46] | 25 (6/24) [10, 47] | | 0.774 |  | 40 (17/42) [26, 57] | 48 (13/27) [29, 68] | 0.211 |
| Negative predictive value | 86 (12/14) [58, 98] | 77 (24/31) [59, 90] | 0.311 |  | 100 (10/10) [69, 100] | 100 (11/11) [72, 100] | 0.999 |  | 100 (3/3) [29, 100] | 83 (5/6) [36, 100] | | 0.279 |  | 90 (19/21) [70, 99] | 83 (30/36) [67, 94] | 0.212 |

Reference: ≥ 70% diameter vessel stenosis detected in quantitative ICA. Data are the results of consensus reading of two readers. Unless otherwise stated, data are percentages, data in parentheses are raw data, and data in brackets are 95% confidence intervals. The 95% confidence intervals were estimated for unclustered data [see reference 31]. ^a^ Data are the area under the curve and data in brackets are 95% confidence intervals. *bpm* beats per minute, *CAD* coronary artery disease, *CTP* computed tomography perfusion, *HSR* halfscan reconstruction, *ICA* invasive coronary angiography, *MSR* multisegment reconstruction

**Supplementary Table 8.** Patient subgroups in per-territory level analysis using ≥ 70% stenosis as reference: diagnostic performance of MSR and HSR of myocardial CTP

|  | **Patients with known CAD** | | |  | **Patients with suspected CAD** | | |  | **Patients with high heart rates  ≥ 75 bpm** | | |  | **Patients with low heart rates  < 75 bpm** | | |
| --- | --- | --- | --- | --- | --- | --- | --- | --- | --- | --- | --- | --- | --- | --- | --- |
| **Reconstruction /**  **performance** | **HSR** | **MSR** | ***p*-value** |  | **HSR** | **MSR** | ***p*-value** |  | **HSR** | **MSR** | ***p*-value** |  | **HSR** | **MSR** | ***p*-value** |
| Area under the curve^a^ | 0.81  [0.73, 0.89] | 0.69  [0.57, 0.81] | 0.021 |  | 0.95  [0.90, 1.00] | 0.73  [0.56, 0.90] | 0.010 |  | 0.85  [0.74, 0.95] | 0.66  [0.43, 0.89] | 0.029 |  | 0.87  [0.80, 0.94] | 0.73  [0.62, 0.84] | 0.007 |
| Sensitivity | 82 (18/22) [60, 95] | 50 (11/22) [28, 72] | 0.023 |  | 100 (10/10) [69, 100] | 60 (6/10) [26, 88] | 0.133 |  | 100 (8/8) [63, 100] | 50 (4/8)  [16, 84] | 0.134 |  | 83 (20/24) [63, 95] | 54 (13/24) [33, 74] | 0.023 |
| Specificity | 68 (131/193) [61, 74] | 80 (154/193) [73, 85] | < 0.001 |  | 81 (54/67) [69, 89] | 79 (53/67) [67, 88] | 0.999 |  | 61 (52/85) [50, 72] | 71 (60/85) [60, 80] | 0.027 |  | 76 (133/175) [69, 82] | 84 (147/175) [78, 89] | 0.011 |
| Positive predictive value | 23 (18/80) [14, 33] | 22 (11/50) [12, 36] | 0.894 |  | 43 (10/23) [23, 66] | 30 (6/20) [12, 54] | 0.049 |  | 20 (8/41) [9, 35] | 14 (4/29)  [4, 32] | 0.220 |  | 32 (20/62) [21, 45] | 32 (13/41) [18, 48] | 0.909 |
| Negative predictive value | 97 (131/135) [93, 99] | 93 (154/165) [88, 97] | 0.017 |  | 100 (54/54) [93, 100] | 93 (53/57) [83, 98] | 0.038 |  | 100 (52/52) [93, 100] | 94 (60/64) [85, 98] | 0.039 |  | 97 (133/137) [93, 99] | 93 (147/158) [88, 96] | 0.012 |

Reference: ≥ 70% diameter vessel stenosis detected in quantitative ICA. Data are the results of consensus reading of two readers. Unless otherwise stated, data are percentages, data in parentheses are raw data, and data in brackets are 95% confidence intervals. The 95% confidence intervals were estimated for unclustered data [see reference 31]. ^a^ Data are the area under the curve and data in brackets are 95% confidence intervals. *bpm* beats per minute, *CAD* coronary artery disease, *CTP* computed tomography perfusion, *HSR* halfscan reconstruction, *ICA* invasive coronary angiography, *MSR* multisegment reconstruction
